# Chiral Recognition of L- and D- Amino Acid by Porphyrin Supramolecular Aggregates

**DOI:** 10.3390/molecules24010084

**Published:** 2018-12-27

**Authors:** Rosalba Randazzo, Massimiliano Gaeta, Chiara Maria Antonietta Gangemi, Maria Elena Fragalà, Roberto Purrello, Alessandro D’Urso

**Affiliations:** Dipartimento di Scienze Chimiche, Università degli Studi di Catania, Viale A. Doria 6, 95125 Catania, Italy; rrandazzo@unict.it (R.R.); mgaeta@unict.it (M.G.); gangemichiara@unict.it (C.M.A.G.); me.fragala@unict.it (M.E.F.)

**Keywords:** amino acid, porphyrin, circular dichroism, supramolecular chirality, self-assembly

## Abstract

We report of the interactions between four amino acids lysine (Lys), arginine (Arg), histidine (His), and phenylalanine (Phe) with the J-aggregates of the protonated 5,10,15,20-tetrakis(4-sulfonatophenyl)-porphyrin H_4_TPPS. Several aspects of these self-assembled systems have been analyzed: (i) the chiral transfer process; (ii) the hierarchical effects leading to the aggregates formation; and, (iii) the influence of the amino acid concentrations on both transferring and storing chiral information. We have demonstrated that the efficient control on the J-aggregates chirality is obtained when all amino acids are tested and that the chirality transfer process is under hierarchical control. Finally, the chiral porphyrin aggregates obtained exhibit strong chiral inertia.

## 1. Introduction

Self-assembly is at the basis of all supramolecular chemistry. It is defined as the spontaneous (not guided) organization of molecules as they obtain larger structures based on a recognition process [1]. Knowing in detail the characteristics of the starting molecules and the interaction types, which acts as a glue for the building of the final aggregate, it is possible to control the features of the final species [2]. Although the driving forces of the self-assembly process are the weak interactions within the structures (electrostatic, π–π stacking, Van der Waals, etc.), the wide network established enables supramolecular assembly with a high degree of stability. The electronic communications between the molecules within the complex induce the transmission of various properties from a single molecule to the entire supramolecular assembly process. The introduction of chirality into the supramolecular species has been widely studied and appears to be one of the most intriguing properties. 

Chirality is a fascinating phenomenon found in nature at all levels. Many biological mechanisms, that are essential to life, involve the use of chiral molecules [3]. In this respect, the chiral recognition of chiral molecules assumes a key role in biotechnology and biochemistry [4,5]. In addition, chirality has applications in numerous scientific fields from medicinal chemistry to material science [6,7,8], and sensing [9,10]. For these reasons, the study on the chirality induction from a molecular assembly to a supramolecular assembly has been an issue of great interest for many years [11]. Chirality transfer mechanisms can be summarized in two potential pathways: (i) as the “dynamic” induction process is under thermodynamic control, if the interactions between the host and the guest are interrupted the induced chirality is lost; and, (ii) the “static” mechanism is under kinetic control and thus chirality becomes an intrinsic property of the new species which is maintained even if the chiral inducer is removed from the solution, exhibiting chiral memory phenomenon [12].

**Scheme 1:**
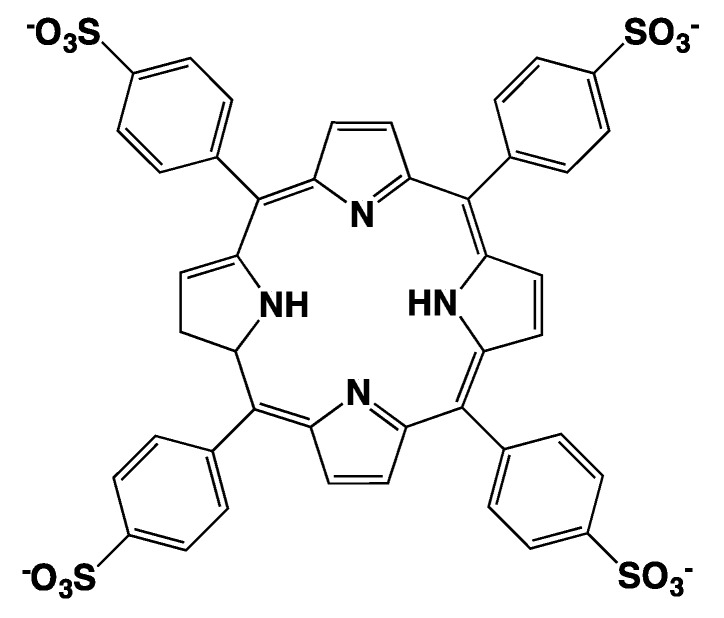
Structural formula of H_2_TPPS porphyrin.

Porphyrins are useful building blocks in the creation of supramolecular multicomponent systems with desired properties owing to their chemical and electronic versatility [13]. Among the porphyrins used to study the chirality transfer processes, the 5,10,15,20-tetrakis(4-sulfonatophenyl)-porphyrin (H_2_TPPS, Scheme 1) is the most intriguing. In specific experimental conditions (pH, ionic strength, concentration, etc.) the protonated form of H_2_TPPS (H_4_TPPS) self-assembles into the H- and J-aggregates with different shapes and sizes [14,15,16,17,18,19,20]. Although H_2_TPPS is not a chiral molecule, the J-type aggregate randomly shows either a positive or negative coupled Circular Dichroism (CD) signal [21]. The origin of this signal remains in discussion within the scientific community and the most accepted hypothesis is that the J-aggregates are inherently chiral and traces of the chiral contaminants shift the 1:1 racemate equilibrium [22,23]. If aggregation is induced in the absence of the chiral compounds while in pure water (ultra-clean conditions; see Experimental Section) no CD signal is detected.

Nevertheless, the J-aggregates can adopt the desired chirality by using several chiral inducers such as cationic polymers [24,25,26,27], metallic complexes [28], dicarboxylic acids [29], nanoparticles [30], exploitation of asymmetric physical fields (e.g., vortexes, temperature gradients, swirling airflow, and magnetic forces) [31,32,33,34], and Circular Polarized Luminescence [35].

Single amino acids have been employed by researchers to transfer chirality to porphyrin’s homo- and hetero-aggregates using either covalent [36,37,38,39,40,41,42,43] or non-covalent approaches [13,44,45]. However, minimal research reports on the chiral properties of the J-aggregates of H_4_TPPS in the presence of amino acids [46,47,48]. In order to investigate the effects of different amino acids on the formation of the H_4_TPPS J-aggregates, we investigated the interactions between four amino acids and H_2_TPPS in a water solution both at neutral and acidic pH. The amino acids selected exhibit distinct isoelectric point IEP and can exploit different weak interactions (electrostatic, π–π stacking, and H-bonding) to influence the J-aggregates formation. By the utilization of UV-vis, resonance light scattering (RLS), and circular dichroism (CD) spectroscopies, we analyzed the chirality transfer process, the hierarchical effect, and the memorization of chirality in order to obtain new knowledge into the formation of supramolecular species.

## 2. Results and Discussion

**Figure 1:**
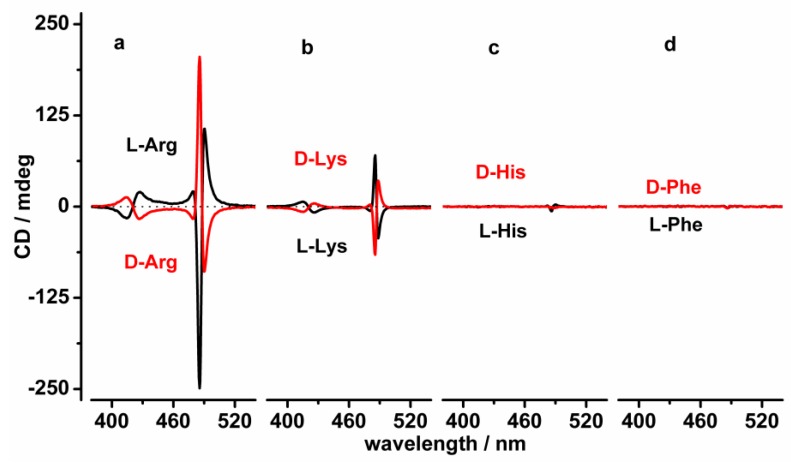
The CD spectra of the J-aggregates obtained with the protocol “acid first” template on: (**a**) 1 mM Arg; (**b**) 1 mM Lys; (**c**) 1 mM His; and, (**d**) 1 mM Phe (black curves represent the L- amino acids and red curves represent the D- amino acids).

Initially, we investigated if all four amino acids act as efficient inducers of chirality to the J-aggregates of H_4_TPPS. The addition of H_2_TPPS (6 µM) to a 1 mM solution of amino acid at pH 2.5 in the presence of NaCl (“acid first” procedure; see material and methods session) results in different behavior depending on the amino acid used. At 1 mM amino acid concentration, in the presence of Arginine (Arg) and Lysine (Lys) only, the CD signal was detected in the absorption region of the porphyrin aggregate, as shown in Figure 1a,b. A mirror image induced CD (iCD) signal is observed for aggregates obtained when using a templating agent of the two enantiomers within the same amino acid. The addition of H_2_TPPS to the acid solution of Histidine (His) and Phenylalanine (Phe), leads to the undetectable CD signal in the absorption region of the J-aggregates, as shown in Figure 1c,d. In order to avoid kinetic interferences all of the solutions are left to equilibrate for one night before performing the CD spectra.

Unexpectedly, Arg and Lys show opposite configuration effects, as shown in Figure 1a,b. Moreover, the inducing effect of Arg was more efficient than the other amino acids (Lys, His, and Phe). These results suggest that the intensity and shape of the induced CD signal of the J-aggregates strongly depend on the type and configuration of the amino acids and, therefore, indicate that the side groups (in terms of size and charge) of the amino acids affect the formation of the supramolecular chiral assembly process.

**Figure 2:**
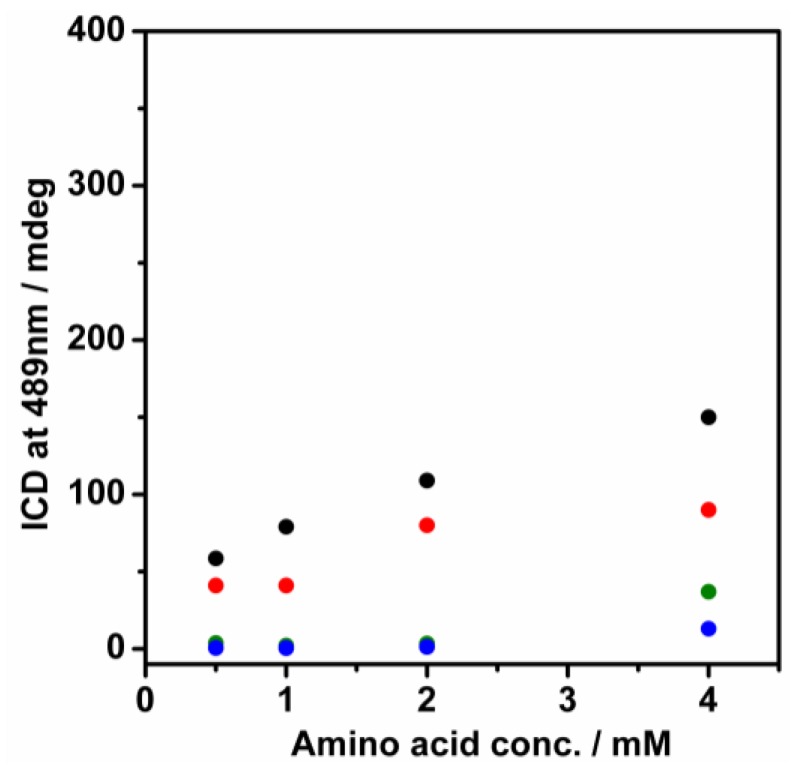
Intensity of the iCD at 489 nm (absolute values) versus the amino acid concentrations. Black dots represent Arg; red dots represent Lys; green dots represent His; and, blue dots represent Phe.

It has been demonstrated that the amino acids form clusters of different shapes and sizes in water solution depending on the concentration used and the dimensions of the clusters are crucial in the chiral induction of porphyrin aggregates [44]. For every amino acid present there should be a distinct concentration threshold. Below this point, there should be no chiral transfer observed owing to the absence of clusters or due to the small size of the clusters. In order to estimate the contribution of the amino acid amount, we performed the same experiments using four different concentrations of amino acids in the solution (0.5 mM; 1 mM; 2 mM; and, 4 mM, as shown in Figure S1). Reporting the CD intensity at 489 nm versus the concentration of amino acid, we determined the minimum amount of amino acid required to induce chirality to H_4_TPPS J-aggregates, as shown in Figure 2. Both Arg and Lys induce evident CD signaling in the J-aggregate absorption region even at 0.5 mM amino acid concentration, with linear trend increasing the concentration. Alternatively, the minimum concentration needed of His and Phe to observe the CD signal in the porphyrin J-aggregate absorption region is 4 mM, as shown in Figure 2.

In order to investigate if our systems were affected by the hierarchical effect, we performed the same experiments shown above by using the second procedure called “acid last”. The hierarchical effect is a time-dependent process which often affects the self-assembly of the supramolecular systems [49]. It has been reported that even self-aggregation of porphyrins in water solution can be governed by the hierarchical effect. By changing the addition in the order of reagents, different outcomes in terms of size, shape, and chirality of the supramolecular assembly can be reached [21,50].

**Figure 3:**
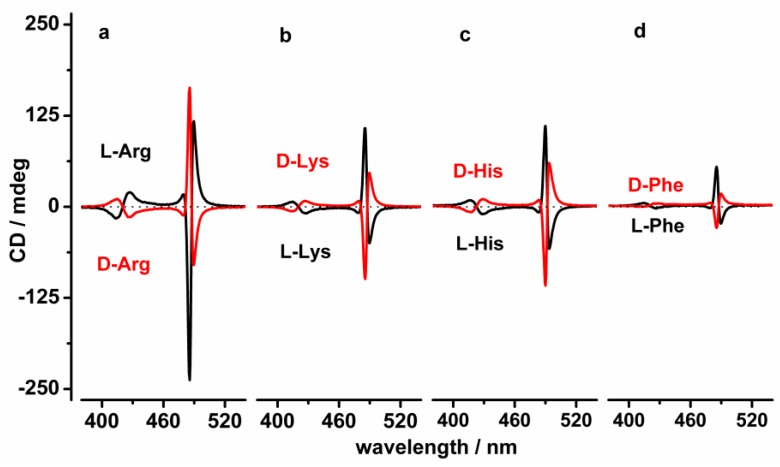
The CD spectra of J-aggregates obtained with the procedure “acid last” template on: (**a**) 1 mM Arg; (**b**) 1 mM Lys; (**c**) 1 mM His; and, (**d**) 1 mM Phe. Black curves represent L- amino acids and red curves represent D- amino acids.

We incubated the amino acid, NaCl, and H_2_TPPS (6 µM) at pH 6.5 for one night and then added HCl to reach pH = 2.5 and subsequently performed the measurements after equilibration the following day. In this context, the possible interactions between the porphyrin compound and the amino acids, established at pH 6.5, could potentially influence the formation of the J-aggregates after protonation. Surprisingly, we did not detect any clear-cut spectroscopic evidence of the interaction between the porphyrin compounds and the amino acids at pH 6.5 (no induced CD signals and no hypochromic effect in the absorption band of the porphyrin was detected), as shown in Figures S2 and S3. The electrostatic interactions are the driving forces behind the formation of the amino acid-(anionic)porphyrin compound and, therefore, amino acids must be positively charged. At pH 6.5, even if the -NH_2_ and the side chains of the amino acids are protonated the -COO^−^ of the amino acid is completely deprotonated which increases the electrostatic repulsions between the negatively charged porphyrins and the amino acids. However, even if there was no evidence of interactions at pH 6.5 the amino acids can transfer the chiral information to the protonated porphyrin aggregates. By decreasing the pH of the solution from 6.5 to 2.5, the formation of the chiral J-type aggregates (whose sign depends on the enantiomer of the amino acid) were detected. According to the results obtained with the “acid first” procedure, positively induced CD coupling with the aggregates template onto D-Lys, D-Phe, D-His, and L-Arg (mirror image was detected using opposite enantiomer) was recorded, as shown in Figure 3.

**Figure 4:**
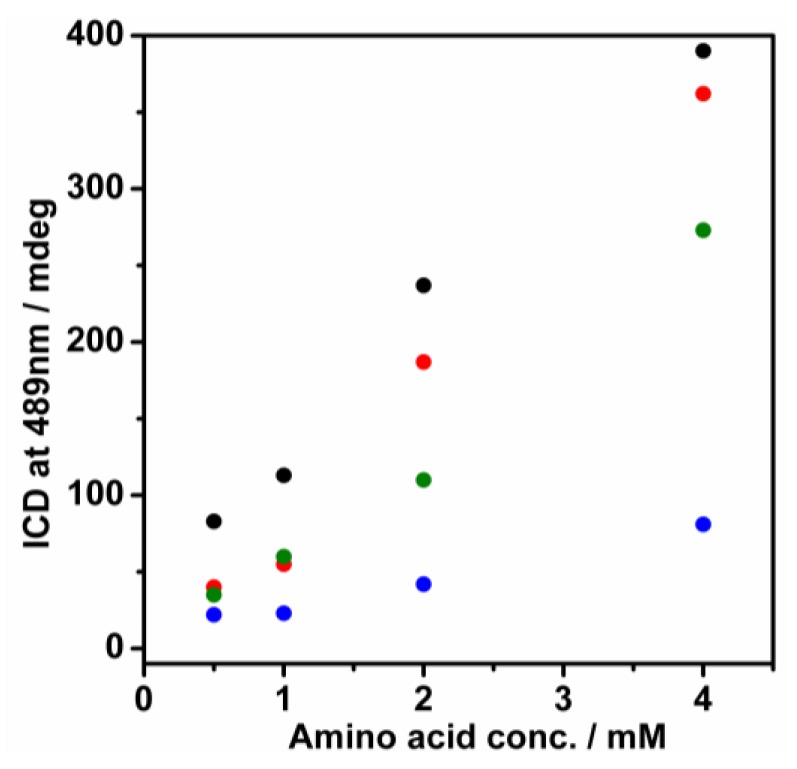
Intensity of the iCD at 489 nm (absolute value) versus the amino acid concentration. Black dots represent Arg; red dots represent Lys; green dots represent His; and, blue dots represent Phe.

Using the “acid last” procedure, we performed experiments operating with different amino acid concentrations ranging from 0.5 mM to 4 mM in order to identify the minimum amount of amino acids required to induce chirality in the porphyrin J-aggregate, as shown in Figure 4 and Figure S4. The experiments confirmed that a 0.5mM solution of Arg, Lys, and His can induce chirality in the H_4_TPPS homo-aggregates. Whereas, Phe has a lower threshold value to transfer chirality in the porphyrin aggregates (1 mM).

Therefore, from the comparison of the induced CD signal intensity of aggregates obtained with both the “acid first” and “acid last” procedures, the results conclude that the intensity of the induced CD signal of the J-aggregates achieved by the “acid last” procedure were highest. These results can be ascribed to the different kinetics of the J-aggregate formation observed for the two distinct procedures. As reported previously [14], in the “acid first” procedure the porphyrin compound is added as the last reagent and the kinetic formation of the J-aggregates is therefore increasingly rapid. The obtained species increases following the chirality of the chiral inducer only if there is a strong interaction between the protonated porphyrins and the amino acids. It is likely that the electrostatic interactions play an important role and indeed, at pH 2.5 all the amino acids used present with positive charges while the protonated form of the porphyrin compound maintains two negative charges. In the “acid last” procedure, the J-aggregates formation is kinetically slower and the protonated porphyrin compounds have more time to form additional order in the aggregates templated onto the chiral amino acids, resulting in a more intense CD signal.

**Figure 5:**
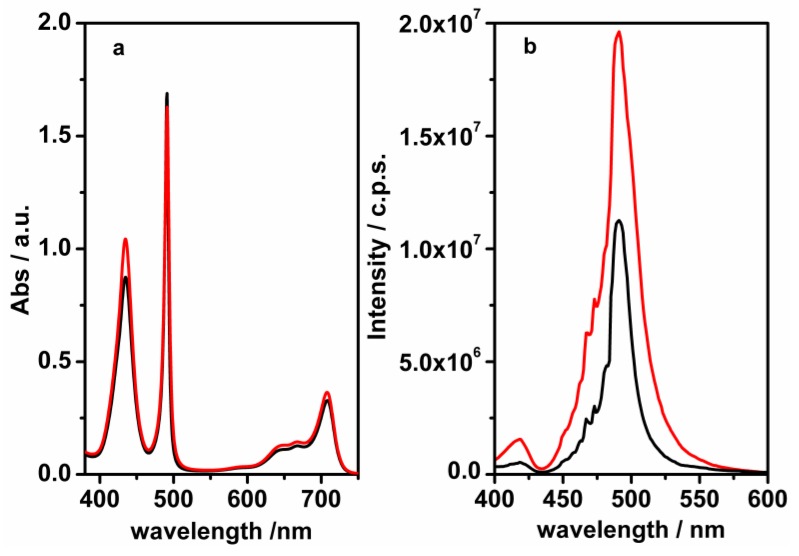
(**a**) UV-vis spectra; and, (**b**) RLS spectra of the solutions of the J-aggregates template on L-Lys 2 mM (black curves represent the “acid first” procedure, and red curves represent the “acid last” procedure).

The difference between the aggregates obtained following the two procedures is more evident when comparing UV, CD, and RLS spectra recorded using L-Lys 4 mM for both procedures, as shown in Figure 5. As shown above (Figure 3), the induced CD signals of the J-aggregates obtained with the “acid last” procedure are more intense and narrower than those recorded when acid is added as the first reagent. Even if UV-vis spectra display the same spectroscopic evidence for the J-aggregates obtained by using both procedures, as shown in Figure 5a, Figures S3 and S5, RLS spectra confirms the formation of the ordered J-aggregates with a strong electronic communication between the porphyrin compounds in the assembly using the “acid last” procedure, as shown in Figure 5b. The RLS signal recorded for the J-aggregates obtained with the “acid first” procedure is less intense than that recorded with the “acid last” procedure.

**Figure 6:**
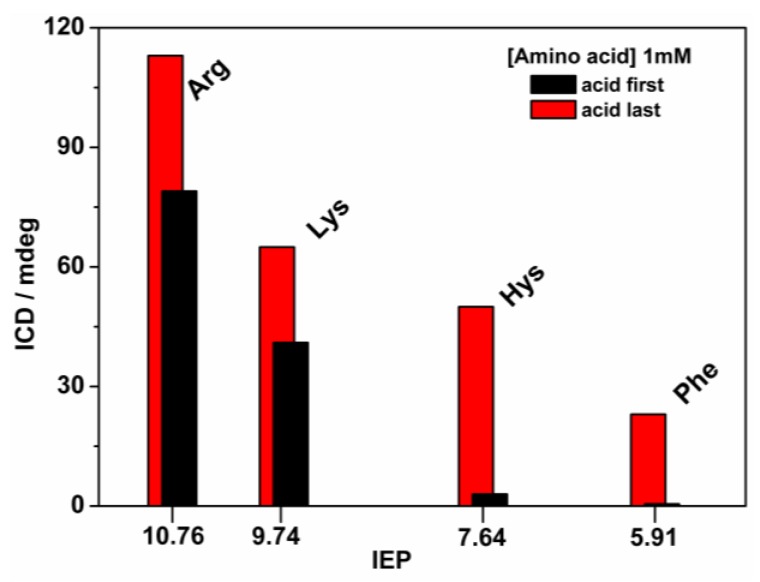
Intensity of the iCD at 489 nm (absolute value) versus the isoelectric point (IEP) of the amino acids for both procedures: “acid first” (represented by the black bars) and “acid last” (represented by the red bars).

Importantly, to find an explanation for the different interactions between the porphyrin compound and the amino acids tested, we correlated the iCD intensity of the J-aggregates (obtained via both procedures) with the amino acid isoelectric points (IEP), as shown in Table S1 and Figure 6. Of note, the high intensity of the J-aggregate induced CD signal is detected for the amino acid with the higher IEP (Arg). Contrarily, we observed that by using a lower intensity of the induced CD signal using Phe the lowest IEP values were obtained, as shown in Figure 6 and Table S1. This behavior is confirmed for all experiments performed, as shown in Figure S6. Considering that in both procedures (“acid first” and “acid last”) the final pH of the solutions after the addition of HCl is 2.5, we can assume that the amino acids with higher IEPs have a large number of positively charged side chains which suggests that the electrostatic interactions are the driving force for the efficient induction of chirality in the J-aggregates of H_4_TPPS.

**Figure 7:**
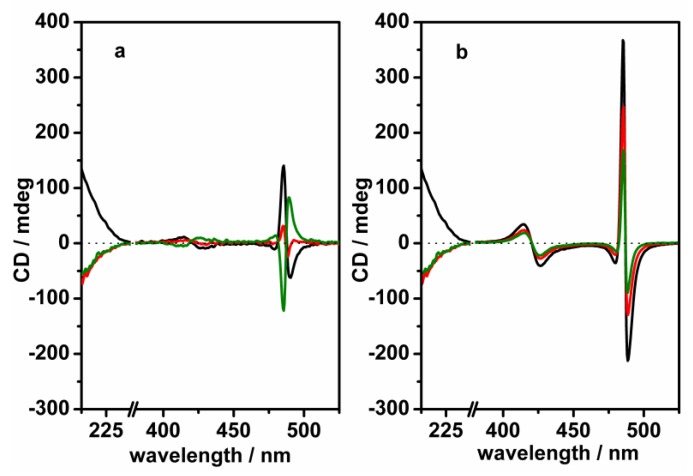
(**a**) CD spectra of the solution of the J-aggregate template on L-Lys 5 mM using the “acid first” procedure before (represented by the black curve), after 24 h (represented by the red curve), and 1 week (represented by the green curve) from the addition of D-Lys 7.5 mM. (**b**) CD spectra of the solution of the J-aggregate template on L-Lys 5 mM using the “acid last” procedure (represented by the black curve), after 24 h in the presence of D-Lys 7.5 mM (represented by the red curve), and after 1 week (represented by the green curve).

Finally, in order to investigate if other types of interactions are involved in the stabilization of these complexes, we tested the chiral inertness of the J-aggregates obtained via both procedures. These experiments evaluate the capabilities of the J-aggregates to store the chiral information transferred by the amino acid. We added an excess of the D- enantiomer of the amino acid to the J-aggregates solution templated onto the L- amino acid (obtained via both procedures). In the case of the “acid first” procedure, after 24 h the induced CD signal drastically changes and indeed, the negative coupling initially observed transforms into a positive coupling following the chiral effect of the amino acid added in excess, as shown in Figure 7a. Contrarily, in the case of the “acid last” procedure, after one week the J-aggregate induced CD signal shows the same sign recorded with the amino acid used as a templating agent and only a reduction of the intensity is observed, as shown in Figure 7b.

These experiments suggest that during the “acid last” procedure large ordered J-aggregates are stabilized by the network of electrostatic and solvophobic interactions that are established between the porphyrin compounds in the assembly process. Therefore, by exploiting the kinetic inertness of the J-aggregates we realized that the chiral supramolecular system can retain its chiral information. Importantly, our research demonstrates that small molecules can be used as a chiral template, thus allowing the memorization of chiral information.

## 3. Materials and Methods

All chemicals were commercially available, used without further purification, and dissolved in ultrapure water obtained from Elga Purlab Flex system by Veolia (Obeivier, France).

The porphyrin H_2_TPPS compound was purchased from Mid-Century (Posen, IL, USA). The H_2_TPPS stock solutions were prepared in ultrapure water and their concentrations (ranging from 10^−4^ M to 2 × 10^−4^ M) were estimated by using the maximum intensity of the Soret band at 412 nm, using an extinction coefficient of 4.8 × 10^5^ M^−1^cm^−1^. The porphyrin solutions were kept in the dark to avoid undesired photochemical reactions. In all experiments the H_2_TPPS concentration was fixed at 6 μM.

The amino acids were purchased from Sigma Aldrich (St. Louis, MO, USA). The stock solutions were prepared by dissolving the exact weight of solid in ultrapure water in order to obtain concentrations ranging from 10^−2^ M to 2 × 10^−2^ M. Absorption and CD measurements were performed on a Jasco V-650 and Jasco J-810 (JASCO, Cremella, Italy), respectively. RLS spectra was recorded on a Fluorolog FL11 Jobin Yvon Horiba instrument (Horiba, Kyoto, Japan). All measurements were performed at room temperature (25 °C) and a 1 cm quartz cuvette was used.

The work solutions were prepared in ultra clean conditions: (i) the operators wore a lab coat, hair cap, gloves, and mask during the preparation of samples; and, (ii) the tips of the pipettes were washed three times with ultrapure water while the vials were treated with an ultrasonic system three times by dipping them in ultra-pure water, before being used. The samples were prepared as follows: (i) in the case of the “acid first” procedure, the needed volume of the amino acid stock solution was diluted in water to reach the desired concentrations (0.5 mM; 1 mM; 2 mM; and, 4 mM); the NaCl (0.3 M) and HCl (pH 2.5) were then added and the porphyrin (H_2_TPPS) was inserted at the opportune volume in order to obtain 6 μM concentration. Finally, the solutions were left to equilibrate for one night prior to recording the spectra; and, (ii) in the “acid last” procedure, the porphyrin compound was mixed with the amino acid and NaCl at a pH of 6.5 then left for one night. HCl was then added as the last reagent in order to reach pH 2.5. The solutions, with all reagents, were left to equilibrate for one night prior to performing any measurements.

## 4. Conclusions

We demonstrated molecular recognition phenomena of amino acids by inducting the formation of supramolecular porphyrin aggregates. By exploiting the hierarchical effects and the kinetic inertness of the porphyrin aggregates, homochiral growth and memorization of chiral information can be achieved. Interestingly, depending on the order of the addition of the compounds in the reaction container, different porphyrin supramolecular structures were achieved and indeed, UV, CD, and RLS measures have confirmed the specific spectroscopic behavior of the J-aggregates prepared with both the “acid first” and “acid last” procedures. The correlation between amino acid IEP and the J-aggregate iCD have allowed us to understand the role of electrostatic interactions in promoting the formation of chiral assembly, however, the kinetic inertness of the J-aggregate has suggested that hydrophobic interactions act as a glue in the porphyrin supramolecular systems. This dichotomy could mark the beginning in the design of a supramolecular device with applications in biotechnology.

## Supplementary Materials

The following are available online at, Figure S1: CD spectra of H_4_TPPS J-aggregates obtained using the procedure *acid first*. Figure S2: CD spectra of Lys after one-night incubation with H_2_TPPS4 solution at pH 6. Figure S3: UV Vis spectra of Lys/H_2_TPPS4 complex preparation using the procedure *acid last*. Figure S4: CD spectra of H_4_TPPS4 J-aggregates obtained using the procedure *acid last*. Figure S5: UV Vis spectra of Lys/H_2_TPPS4 complex preparation using the procedure *acid first*. Table S1: pKa values and structures of the amino acids. Figure S6: ICD vs IEP for each amino acid with both procedures’ *acid first* and *acid last*.
