# Supplementary material for: Chiral Recognition of L- and D- Amino Acid by Porphyrin Supramolecular Aggregates
Source: Molecules. 2018 Dec 27;24(1):84. doi: 10.3390/molecules24010084 (PMC6337589; doi:10.3390/molecules24010084)

Supplementary data

Chiral recognition of L- and D- amino acid by porphyrin supramolecular aggregates

Rosalba Randazzo, Massimiliano Gaeta, Chiara M. A. Gangemi, Maria Elena Fragalà, Roberto Purrello,* and Alessandro D'Urso,*

[1] Dipartimento di Scienze Chimiche, Università degli Studi di Catania, Viale A. Doria 6, 95125 Catania, Italy

* Correspondence: adurso@unict.it; rpurrello@unict.it; Tel.: +39-095-738-5095

Figure S1: CD spectra of H_4TPPS J-aggregates obtained using the procedure *acid first*

Figure S2: CD spectra of Lys after one night incubation with H_2TPPS4 solution at pH 6

Figure S3: UV Vis spectra of Lys/ H_2TPPS complex preparation using the procedure *acid last*

Figure S4: CD spectra of H_4TPPS J-aggregates obtained using the procedure *acid last*

Figure S5: UV Vis spectra of Lys/ H_2TPPS complex preparation using the procedure *acid first*

Table S1. pKa values and structures of the amino acids.

Figure S6: ICD vs IEP for each amino acid with both procedures *acid first* and *acid last*

Figure S1: CD spectra of H_4TPPS J-aggregates obtained using the protocol *acid first* at different amino acids concentrations (black curves 0.5mM, red curves 1mM, green curves 2mM and blue curves 4mM). a) L-Arg, b) L-Lys, c)L-His and d) L-Phe. The same general behaviour (with mirror image spectra) was observed using D- amino acids.

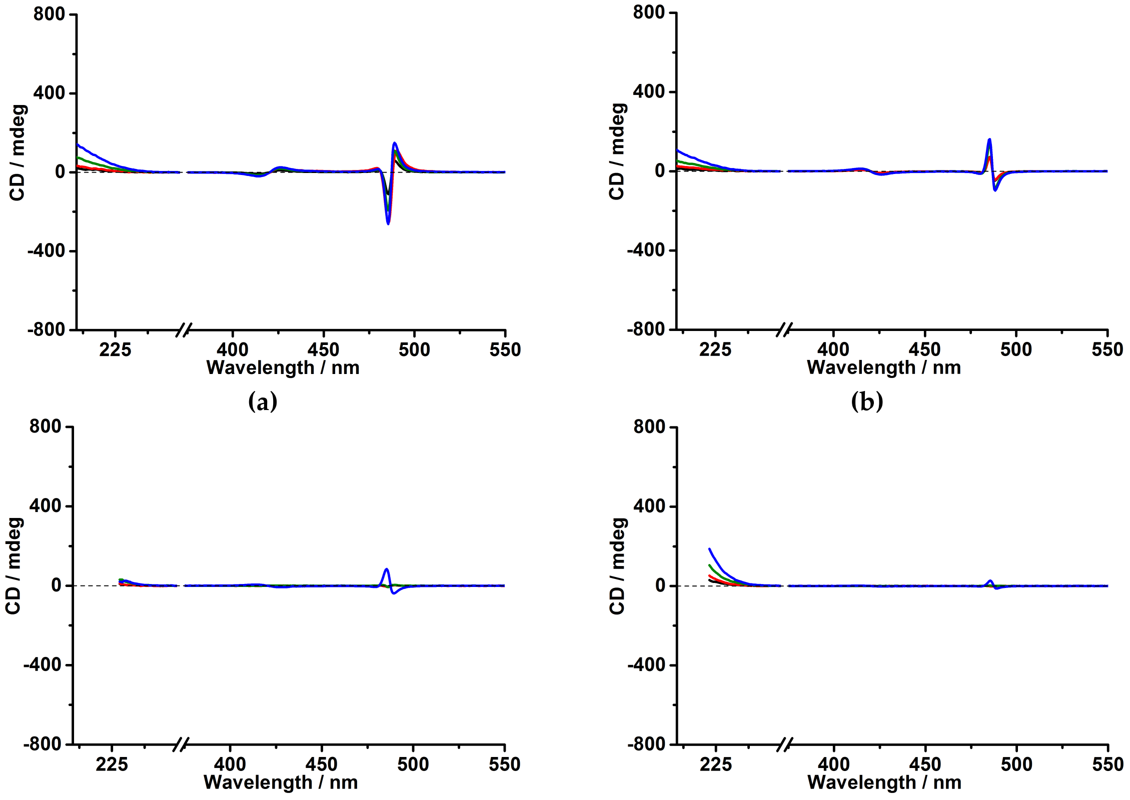

(a) (b) (c) (d)

Figure S2 : CD spectra of Lys 2mM in NaCl 0.3M solution at pH 6 after one night incubation with H_2TPPS 6μM (L-Lys black curve, D-Lys red curve). Same results was observed for all amino acids investigated

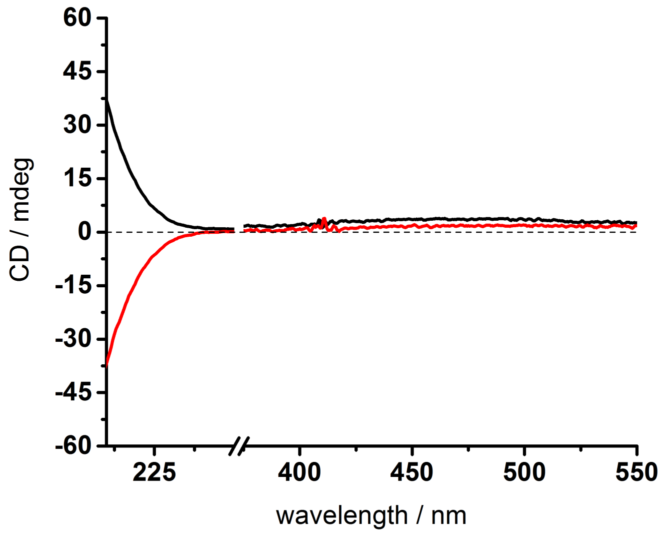

Figure S3: UV Vis spectra of Lys 2mM in NaCl 0.3M solution at pH 6.5 (black curve), after one night incubation with H_2TPPS 6μM (red curve), then after addition of HCl to reach pH 2.5 (green curve) and after 24 hours (blue curve); *acid last* procedure. For comparison UV Vis spectrum of H_2TPPS 6μM solution in NaCl 0.3 M at pH 6.5 is reported (dashed curve).

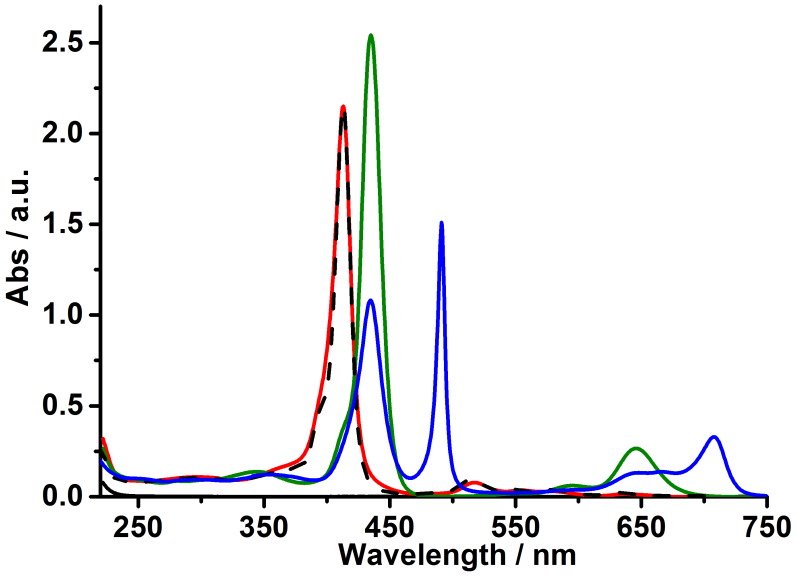

Figure S4: CD spectra of H_4TPPS J-aggregates obtained using the procedure *acid last* at different amino acids concentrations (black curves 0.5mM, red curves 1mM, green curves 2mM and blue curves 4mM. a) L-Arg; b) L-Lys; c) L-His and d) L-Phe. The same general behaviour (with mirror image spectra) was observed using D- amino acids.

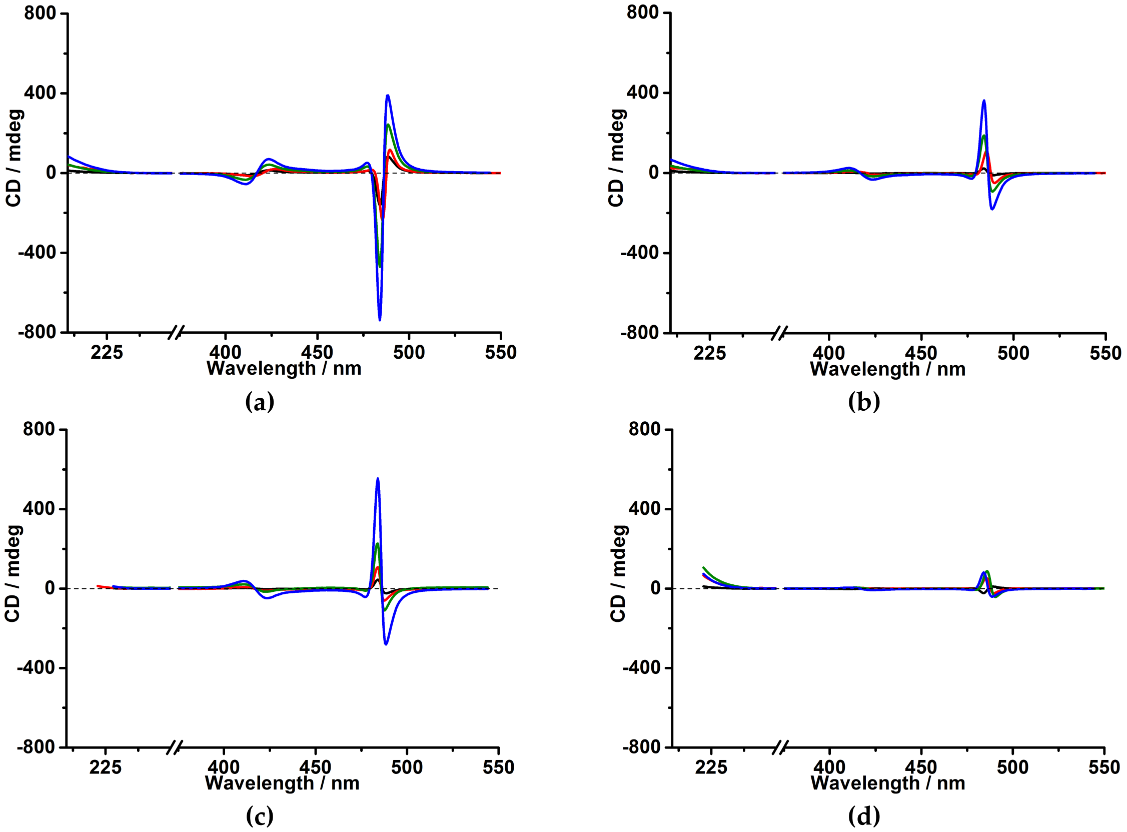

Figure S5: a) Uv-Vis spectra of Lys 2mM in NaCl 0.3 M at pH 2.5 (black curve), immediately after the addition of H_2TPPS 6μM (red curve) and after 24 hours (green curve); *acid first* procedure. For comparison the UV Vis spectrum of H_2TPPS 6μM in NaCl 0.3M at pH 2.5 is reported (dashed curve).

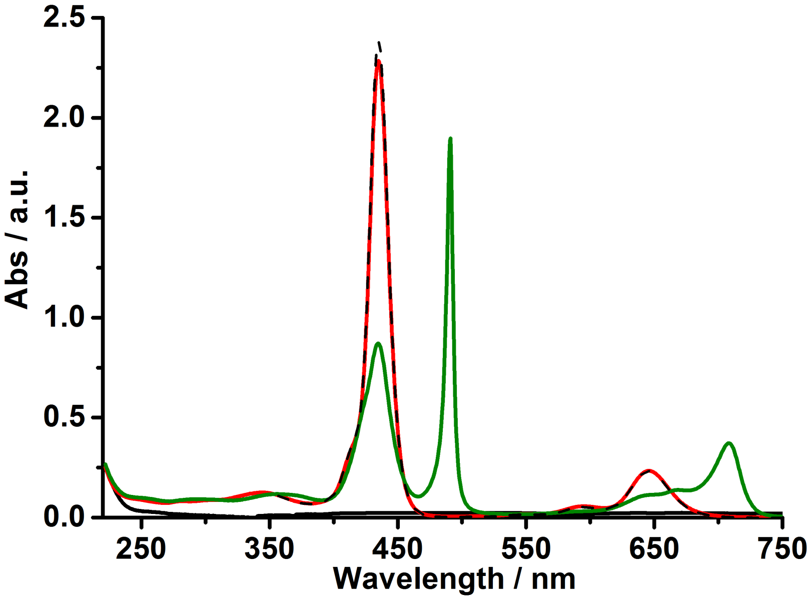

(a)

Table S1. pKa values of the amino acids[a]

Amino acid		pK_a α-CO_2H	pK_a $\alpha-NH_3$	pK_a side chain	IEP
Arginine	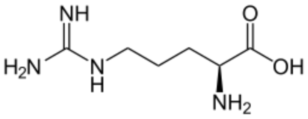	2.01	9.04	12.48	10.76
Lysine	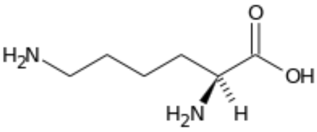	2.18	8.95	10.53	9.74
Histidine	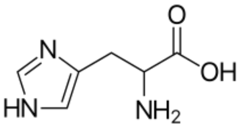	1.77	9.18	6.10	7.64
Phenylalanine	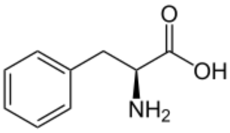	2.58	9.24	-	5.91

[a] W. H. Brown, C. S. Foote, B. L. Iverson, E. V. Anslyn Organic Chemistry Sixth Edition, Brooks/Cole, USA 2012, p.1102

Figure S6: Induced CD signal at 489 nm of J-aggregate/amino acid complexes *vs* Isoelectric points (IEP). Each bar at the same IEP indicates different concentration of amino acid (4mM wine and dark blue bars; 2mM red and blue bars; 1mM magenta and dark cyan bars; 0.5mM pink and cyan bars). The two set of colours indicate the two procedures used: *acid last* (wine, red, magenta and pink bars) and *acid first* (dark blue, blue, dark cyan and cyan bars)

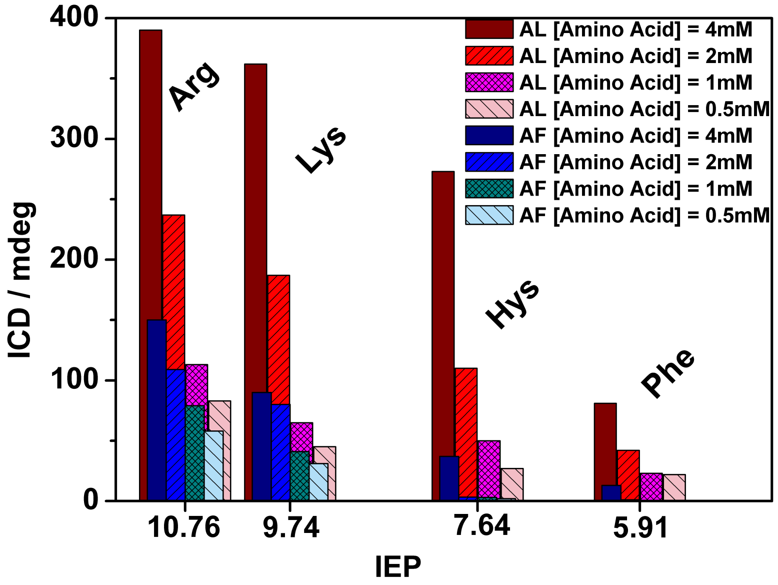

Supplement: Supplementary file 1 [file molecules-24-00084-s001.pdf]
